# Systematic identification and integrative analysis of novel genes expressed specifically or predominantly in mouse epididymis

**DOI:** 10.1186/1471-2164-7-314

**Published:** 2006-12-13

**Authors:** Jungsu Oh, Jiae Lee, Jong-Min Woo, Eunyoung Choi, Inju Park, Cecil Han, Namhoe Baek, Hoyong Lee, Do Han Kim, Chunghee Cho

**Affiliations:** 1 Department of Life Science, Gwangju Institute of Science and Technology, Gwangju 500-712, Korea

## Abstract

**Background:**

Maturation of spermatozoa, including development of motility and the ability to fertilize the oocyte, occurs during transit through the microenvironment of the epididymis. Comprehensive understanding of sperm maturation requires identification and characterization of unique genes expressed in the epididymis.

**Results:**

We systematically identified 32 novel genes with epididymis-specific or -predominant expression in the mouse epididymis UniGene library, containing 1505 gene-oriented transcript clusters, by *in silico *and *in vitro *analyses. The Northern blot analysis revealed various characteristics of the genes at the transcript level, such as expression level, size and the presence of isoform. We found that expression of the half of the genes is regulated by androgens. Further expression analyses demonstrated that the novel genes are region-specific and developmentally regulated. Computational analysis showed that 15 of the genes lack human orthologues, suggesting their implication in male reproduction unique to the mouse. A number of the novel genes are putative epididymal protease inhibitors or β-defensins. We also found that six of the genes have secretory activity, indicating that they may interact with sperm and have functional roles in sperm maturation.

**Conclusion:**

We identified and characterized 32 novel epididymis-specific or -predominant genes by an integrative approach. Our study is unique in the aspect of systematic identification of novel epididymal genes and should be a firm basis for future investigation into molecular mechanisms underlying sperm maturation in the epididymis.

## Background

The mammalian epididymis is a segmented organ comprised of a single highly convoluted tubule divided into four regions: the initial segment, caput, corpus, and cauda regions. As sperm produced in the testis pass through the epididymis, they undergo sequential, marked changes to develop motility and the ability to fertilize an egg [1,2]. Sperm are transcriptionally and translationally inactive. Therefore, post-testicular maturation of sperm is not under the control of the germinal genome but rather it is mediated by factors within the lumen of the epididymis. The contents of the epididymal lumen are constantly changing due to ion transport across the epithelium and protein secretion into the epididymal lumen. Some of these proteins are found only in certain regions (i.e., the initial segment, caput, corpus, or cauda) and their expression is regulated by androgens or testicular factors [3-5]. Efforts have been made to identify the genes involved in sperm maturation during epididymal transit. Some proteins that are secreted into the epididymal lumen and which are believed to be crucial for sperm maturation have been characterized and shown to bind to the sperm surface membrane, but many remain unknown [6-10].

Recent high-throughput genomics projects have focused on the identification of cell- and tissue-specific transcriptomes that are expected to provide important insights into biological processes. Characterization of expressed sequence tags (ESTs) derived from cDNA libraries has led to the discovery of novel genes with tissue-specific expression profiles. Currently, the largest and most widely used EST database is UniGene, which automatically partitions GenBank sequences into non-redundant sets of gene-oriented clusters, so each UniGene cluster contains sequences that represent a unique gene [11]. Each cluster also contains related information such as the dbEST cDNA library from which the sequence was derived. Details of dbEST library construction almost invariably contain information about the tissue from which the library was constructed. As a result, ESTs in UniGene are individually linked to their tissue of origin through their dbEST library ID number. These links provide a simple method for identifying ESTs with increased expression in specified dbEST libraries. Thus, the UniGene databases combined with other computational bioinformatics databases provide a large amount of information to predict the tissue specificity of gene expression, genomic nature, and the putative structure and function of novel gene products.

Comprehensive understanding of epididymal function in sperm maturation requires the identification and functional characterization of epididymis-specific genes, because sperm maturation in the epididymis is a highly specific process that does not occur in any other tissues. In this study, we identified several novel epididymal genes using the epididymis UniGene library. The genes were initially identified by *in silico *analysis and their transcript characteristics, region-specific expression, postnatal expression, and hormonal regulation, and characteristics of the expressed proteins were characterized *in vitro*. Our results demonstrate a tool for identifying genes that may have a crucial role in sperm maturation in the epididymis and that could be used to identify new targets for the development of male contraceptive or infertility treatments.

## Results

### The epididymis UniGene library and *in silico *selection of novel gene candidates with epididymis-specific or -predominant expression

To identify putative epididymis-specific novel genes, we analyzed the epididymis library (Library 2606) deposited in the UniGene data base at the NCBI. At the start of our study (September 2004), the epididymis library contained 1505 UniGene entries. This library was used for an *in silico *search to identify epididymis-specific novel genes according to four criteria: (i) genes previously named or assigned with potential functions were counted as known genes, and unnamed genes with unknown or unassigned function were considered as unknown or novel genes; (ii) UniGene entries composed of a single EST were excluded from the present study as they are likely to be expressed in the tissue at a low level; (iii) if all ESTs of a given gene were epididymal or the number of epididymal ESTs of a gene was much higher than that of non-epididymal ESTs, the gene was considered to be a putative epididymis-specific gene; (iv) if more than half of the ESTs of a particular gene were found in the epididymis, but were also found in certain other tissues (less than three tissues and excluding female organs, such as eggs, ovaries, and mammary glands), the gene was considered to be a putative epididymis-predominant gene. As a result, we identified 409 gene entries with two and more EST copies, and of these 205 represented known genes and 204 represented unknown genes (Table 1 and Additional data files 1 and 2). It should be noted that some of the unknown genes have been annotated or named in updates of the UniGene database during the course of our study. The *in silico *tissue distribution was analyzed by comparing the numbers of ESTs in UniGene libraries of different tissues, and demonstrated that most of the known and unknown genes are widely expressed and relatively few genes are epididymis-specific or -predominant. However, the number of epididymis-specific or -predominant genes in the unknown gene category was much greater than in the known gene category. This indicates that the UniGene epididymis library is a good source for identifying putative epididymis-specific novel genes and that, although several epididymis-specific genes have been characterized in the past few years, many of the epididymis-specific genes have not yet been characterized. In the present study, we selected 83 unknown genes with epididymis-specific or -predominant expression for analysis (Table 1 and Additional data file 2).

### Authenticity of novel genes with epididymis-specific or -predominant expression

**Figure 1:**
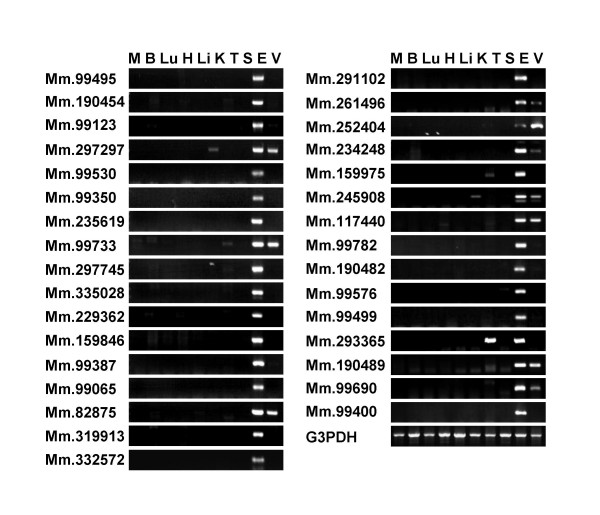
Tissue distribution of the genes by RT-PCR analysis in various tissues of adult male mice. All of the genes were specifically or predominantly expressed in the epididymis. Estimation of intensity of bands amplified using primers specific to the glyceraldehyde-3-phosphate dehydrogenase (G3PDH) gene indicates the equivalent amounts of cDNA template in each tissue. M, skeletal muscle; B, brain; Lu, lung; H, heart; Li, liver; K, kidney; T, testis; S, spleen; E, epididymis; V, vas deferens.

To determine whether the candidates selected from the UniGene library are genuine novel genes with epididymis-specific or -predominant expression, we performed *in silico *analyses and various expression analyses. Analysis of the amino acid sequences deduced from the cDNAs showed that the open reading frames of 42 of the 83 genes were possible encoding sequences, whereas the open reading frames of 41 candidates were too short (less than 5 kDa) or contained unreliable coding regions (e.g., a single coding exon in more than three noncoding exons, or frameshift or stop codons within potentially functional domains). Thus, these 41 candidates were eliminated from further analysis. Reverse transcription-polymerase chain reaction (RT-PCR) analysis showed that 35 of the 42 candidates were expressed with the predicted molecular sizes in the epididymis, whereas the other seven candidates were not detected in the epididymis. It should be noted that the reaction was designed to be similar for all candidate genes and all reaction conditions were the same. Tissue distribution of the 35 candidates was investigated by PCR using cDNAs from various tissues (Table 2). Thirty two of the 35 genes were found to be epididymis-specific or -predominant (Figure 1). Taken together, analyses of the 83 potential genes identified 32 coding genes with epididymis-specific or -predominant expression. Thus, these 32 genes were analyzed further (Table 2).

### Transcript analysis and genomic characterization

**Figure 2:**
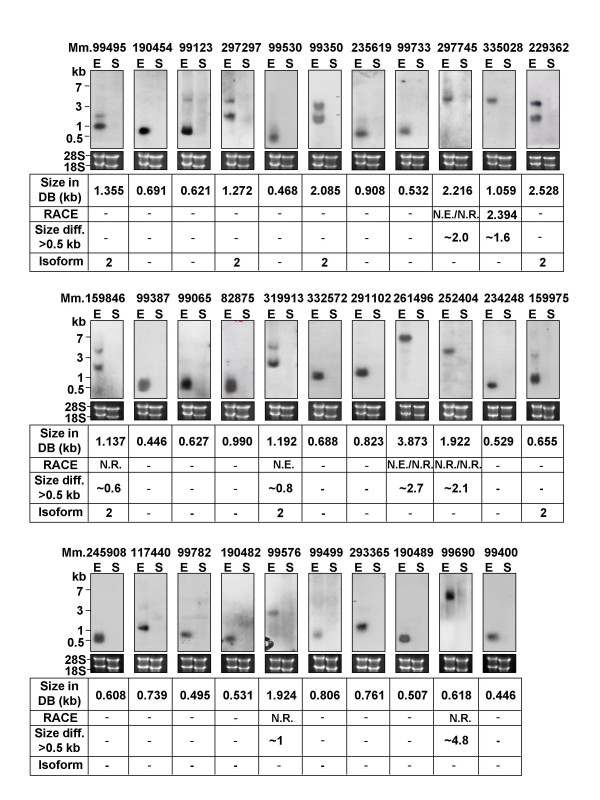
Transcript analysis by Northern-blot hybridization. Total RNA from epididymis (E) and spleen (S) were hybridized with cDNA probes of the genes. Agarose gels were stained with ethidium bromide to visualize 28S and 18S RNAs as a control to ensure loading of the same amount of RNA in each lane. There were significant differences in transcript size between the Northern blots and the UniGene predictions for eight genes, and these genes were subjected to RACE. This resulted in an additional new sequence for a gene (Mm.335028). Transcript sizes from known sequences (UniGene database and RACE), transcripts with significant differences in size between the Northern blots and cDNA sequences, and transcripts with isoforms are indicated below the blots. In the RACE analysis, genes with results in which transcript sequences were not extended (N.E.) or no result (N.R.), i.e. no amplification of transcript sequence in the analysis, are indicated. In the case of results from both 5'- and 3'-RACE in a given gene, the results are shown as 5'/3'.

**Figure 3:**
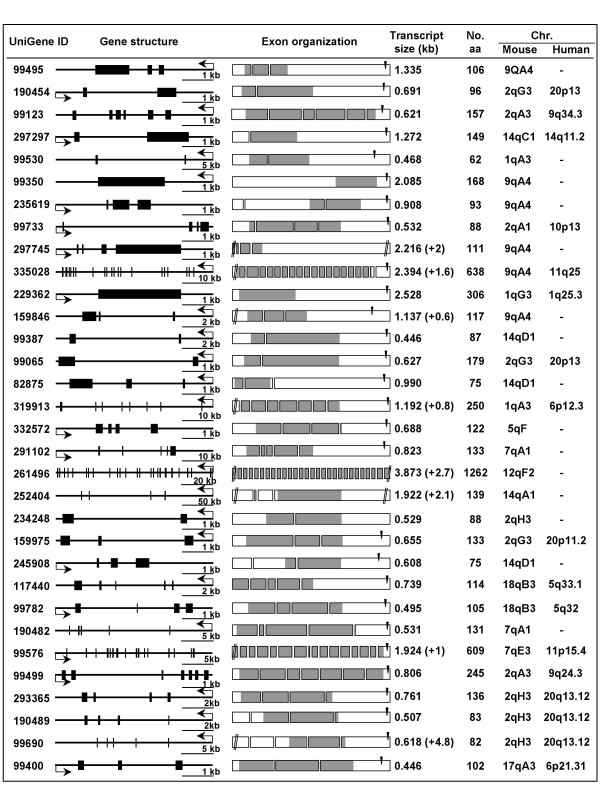
Genomic and transcript characteristics of the novel genes. Gene structure and exon organization were determined by genome database searches. In the gene structure, vertical bars and connecting horizontal lines represent the position of exons and introns, respectively. The orientation of each gene is indicated by a broken arrow. In the exon organization, diagonal lines represent additional unknown sequences. Coding regions were determined by selecting the longest open reading frames deduced from the cDNA sequences, and the predicted coding regions are shaded. The position of the poly(A) signal is marked by filled arrowheads. The calculated transcript sizes are summarized from the recent UniGene databases and the results from the Northern blots shown in Figure. 3. The numbers of amino acids (No. aa) corresponding to the predicted coding regions are listed. Chromosomal locations were determined by searches of the assembled human (UCSC Build 36.1) and mouse (UCSC Build 36) genomes.

To determine the expression levels and transcript sizes of the 32 genes, we performed Northern-blot analysis (Figure 2). For all of the genes, significant amounts of signal were detected in the RNA samples from the epididymis, but not in the samples from the spleen, which were used as a negative control. This result is consistent with the RT-PCR tissue-distribution result (Figure 1) and further demonstrates the abundant expression of the genes in the epididymis. The sizes of the epididymal transcripts ranged from 0.5 kb (UniGene identifier Mm.99530) to 6.6 kb (Mm.261496), and some of these genes (Mm.99495, Mm.297297, Mm.99350, Mm.229362, Mm.159846, Mm.319913, and Mm.159975) produce multiple transcripts of different sizes, which suggests that multiple transcript isoforms are produced by alternative splicing (Figure 2). Considering the polyadenylation tail and 5' or 3' untranslated regions (UTRs), transcript sizes estimated from the UniGene database (based on the GenBank ID in Table 2) were similar to those determined by Northern-blot analysis for most of the genes. Marked differences in transcript size (>0.5 kb) between the Northern blots and the database sequences were found for eight genes, suggesting the presence of additional transcript sequences in these genes. We performed 5' rapid amplification of cDNA ends (RACE) analysis to determine the full-length transcript sequences of these genes. It should noted that 3'-RACE was also performed for three of the eight genes, because no poly(A) signals and sequences were found for these genes in the database (Figure 3). The transcript sequence of Mm.335028 increased from 1.059 to 2.394 kb (GenBank accession number DQ664197), changing the coding sequences. However, extended sequences were not obtained for the other seven genes from the RACE analysis (Figure 2). Taken together, the transcript sequences for at least 24 genes can be regarded as full-length cDNAs or sequences containing entire cDNA sequences. Estimated transcript sizes based on the Northern-blot results and the most recent UniGene database entry are summarized in Figures 2 and 3.

To characterize the novel genes, we performed genome database searches with the transcript sequences. Figure 3 shows structures, exon organization, and chromosomal locations of the genes. The sizes of the genes range from 1.3 to 130 kb. The number of exons in the genes also varies, ranging from a single-exon gene to a 28-exon gene. The novel genes were found to be widely distributed on mouse chromosomes and 17 of these novel genes have human orthologues in the regions of conserved synteny between mice and humans.

### Regulation of gene expression

**Figure 4:**
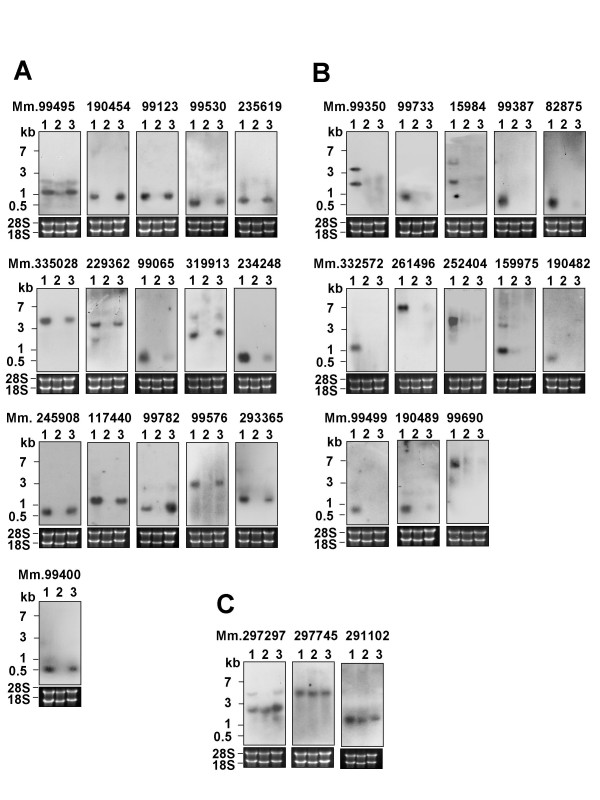
Hormonal regulation of gene expression. Using epididymides from wild-type mice (lane 1) and from castrated mice treated with oil (lane 2) or dihydrotestosterone (lane 3), Northern-blot analysis was performed as described in Fig. 2. Genes were divided into three groups based on whether their expression was regulated by androgen or testicular factors: androgen-dependent **(A)**, testicular factor-dependent **(B)**, and androgen- and testicular factor-independent gene expression **(C)**.

To investigate the transcription regulation of the 32 genes, we analyzed the expression of the genes in gonadectomized mice. We performed Northern-blot analysis using total RNAs isolated from the epididymides of sham-operated (wild-type control) mice, mice that had been bilaterally castrated and reared for 1 week, and mice that had been bilaterally castrated, reared for 1 week then treated with dihydrotestosterone (DHT). The expression of 16 genes stopped after castration, whereas DHT treatment retained the expression partially or completely (Figure 4A). The remaining 16 genes showed an androgen-independent expression pattern. The expression levels of 13 of these genes were downregulated by castration and did not increase after DHT treatment, indicating that the expression of these genes is highly dependent on testicular factors rather than androgens (Figure 4B). The expression of the other three genes did not change notably with castration and DHT treatment, indicating that they are constitutively expressed in the epididymis regardless of the presence of androgen or testicular factors (Figure 4C).

### Regional and developmental expression profile of novel genes

**Figure 5:**
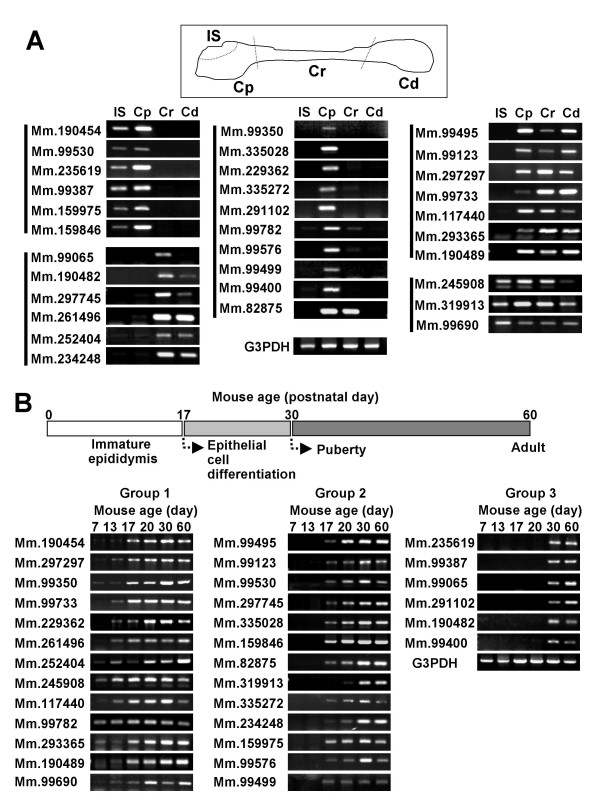
Region-specific and developmental expression profile of novel genes by RT-PCR. **(A) **Region-dependent expression of the novel genes in the epididymis. The schematic organization of the mouse epididymis is shown at the top, highlighting the initial segment, caput, corpus, and cauda regions. RT-PCR analysis was performed with cDNAs prepared from four different regions of epididymis. The house-keeping gene G3PDH was used as a control to normalize the template input. The genes were divided into 5 different groups, based on regional expression pattern and each group is indicated by a vertical bar. IS, initial segment; Cp, caput; Cr; corpus; Cd, cauda. **(B) **Postnatal developmental expression of the novel genes. Schematic diagram of the postnatal development of the epididymis is shown, highlighting the epithelial cell differentiation stage. RT-PCR was performed with cDNAs prepared from epididymides of mice of different ages. Genes were divided into three groups based on the stage of development at which they were expressed: before epithelial cell differentiation (Group I), at epithelial cell differentiation (Group II), and at puberty (Group III). Estimation of intensity of bands amplified using primers specific to the G3PDH gene indicates the equivalent amounts of cDNA template at each stage. The numbers above the lanes indicate days after birth.

Many epididymal genes are expressed in specific regions of the epididymis (the initial segment, caput, corpus, or cauda) [3,4]. Thus, to investigate the region-specific expression of 32 candidate genes, we performed RT-PCR on samples from the four different regions of the epididymis. All of the genes were expressed in at least one region of the epididymis and could be divided into 5 groups based on expression pattern (Figure 5A). The expression of half of the genes was greater in the proximal region of the epididymis, which secretes proteins at a higher rate than other regions and starts the maturational changes, suggesting that these genes have a role in sperm maturation and fertility.

We next investigated the developmental expression pattern of the 32 genes (Figure 5B). RT-PCR analysis on epididymides from mice of different ages demonstrated that 13 genes were expressed early in development (Group 1), during the first few days after birth, whereas another 13 genes were only detectable in mice aged at least 17 days (Group 2), which corresponds to the stage of epithelial cell differentiation. The remaining six genes were only detected in mice aged at least 30 days (Group 3), implying a close relationship between gene expression and puberty [12,13]. These results suggest that many of the novel genes are expressed in epithelial cells of the epididymis where active secretion occurs and has an important role in sperm maturation.

### Analysis of protein characteristics

**Figure 6:**
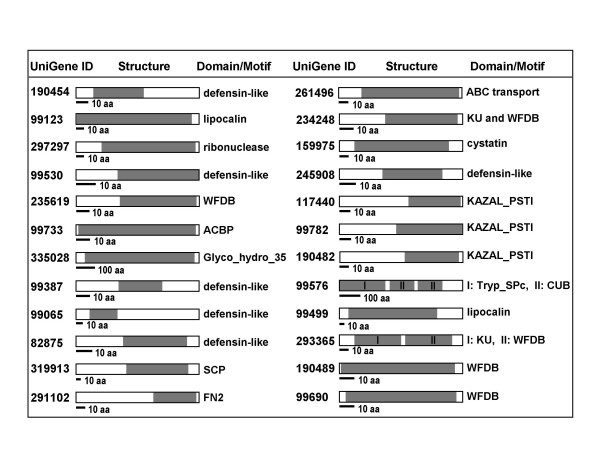
Putative domains and motifs in proteins encoded by the novel genes. The predicted amino acid sequences of the novel genes were analyzed using various bioinformatics tools (see Materials and Methods) and genes containing putative domains or motifs are listed. The proteins are indicated by boxes and, the putative domains or motifs are shaded. The size of the scale bar is shown as number of amino acids (aa) below each protein. Domain/motif abbreviations are as follows: WFDB, whey-acid-protein four-disulfide binding core domain; ACBP, acyl-CoA binding protein; Glyco_hydro_35, Glycosyl hydrolases family 35; SCP, sperm coating protein-like extracellular protein; FN2, type II fibronectin collagen-binding domain; ABC transport, ATP binding cassette transport; KU, BPTI (bovine pancreatic trypsin inhibitor)/Kunitz family of serine protease inhibitors; KAZAL_PSTI, Kazal-type pancreatic secretory trypsin inhibitors (PSTI) and related proteins; Tryp_SPc, trypsin-like serine protease; CUB, CUB domain.

To gain an insight into the structures and functions of proteins expressed from the novel genes, a protein-coding region in each gene was defined by selecting the longest amino-acid-coding sequence, which terminates before a polyadenylation signal (if one is present), and these amino acid sequences were subjected to protein database searches. For most of the genes, the predicted coding regions are considered to be accurate. The exceptions are the eight genes whose transcripts are known to be significantly smaller in size than indicated by the Northern blots (Figures 2 and 3). Nevertheless, it should be noted that all of these genes contain complete coding sequences, suggesting that 5' or 3' UTR sequences are responsible for the size discrepancy between the Northern-blot results and the UniGene database. Analysis of the amino acid sequences defined from the cDNAs by BLAST search revealed that eight genes contained conserved domains characteristic of protease inhibitors such as the Kazal-type serine protease inhibitor domain (Mm.117440, Mm.99782, and Mm.190482) or the whey-acid-protein (WAP) four-disulfide core domain (Mm.235619, Mm.234248, Mm.293365, Mm.190489, and Mm.99690). Furthermore, an additional six genes were found to contain conserved cysteine residues typical of β-defensins (Mm.190454, Mm.99530, Mm.99387, Mm.99065, Mm.82875, and Mm.245908) (Figure 6) [14,15].

**Figure 7:**
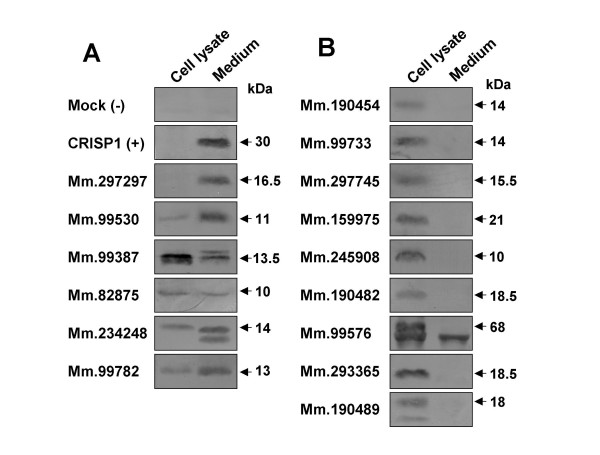
Secretion of proteins encoded by the novel genes. COS-7 cells were transfected with pcDNA3.1-UniGene-myc/His. After 48 hours, UniGene-myc/His were immunoprecipitated with anti-myc mAb from the culture medium and the cell lysates, and then subjected to Western-blot analysis using α-myc. Proteins were divided into two groups based on their secretion profile: secretory **(A) **and intracellular **(B) **proteins. Vector alone (Mock) and cysteine-rich secretory protein 1 (CRISP1) were used as a negative control and secretion marker, respectively. The lower bands in the immunoblot of Mm.99576 represent immunoglobulin G (IgG) heavy chain. The arrow indicates the molecular weight of the each protein.

It is important to note that epididymal proteins must be secreted to interact with sperm either directly or indirectly. Thus, to investigate if novel proteins are secreted, to further confirm the authenticity of the novel genes, and to determine the sizes of novel proteins expressed in mammalian cells, COS-7 cells were transiently transfected with a pcDNA3.1-myc/His plasmid expressing the 32 novel proteins with a myc/His epitope tag at the carboxy terminus. An immunoblot analysis showed that 15 of the 32 genes were relatively well expressed in COS-7 cells, and the expressed proteins were of the sizes predicted from the cDNA sequences (Figure 7). By contrast, 17 of the 32 genes were not expressed, indicating that the expression of these proteins is highly transient, very low or delayed, vulnerable to the endogenous protease, or toxic to the cells [16]. Of the 15 expressed proteins, six proteins were detected in the culture media (Figure 7A), whereas the remaining nine proteins were detected within the cells (Figure 7B). Interestingly, two of the secreted proteins (Mm.99387 and Mm.234248) were post-translationally modified after secretion, potentially by processes such as glycosylation, phosphorylation, or enzymatic digestion. It should be noted that the six proteins found to be secreted may interact with sperm in the epididymis, playing important roles in sperm maturation or fertility.

## Discussion

In the present study, we identified and characterized 32 novel epididymis-specific or -predominant genes by *in silico *and *in vitro *approaches, providing comprehensive information about the genes. We initially selected these genes by analyzing the epididymis UniGene library. Currently, UniGene is the largest and most widely used EST database and contains a large amount of unanalyzed information. Thus, *in silico *gene identification and analysis is becoming a rapidly expanding and powerful tool of modern molecular biology, and it has been successfully used in several studies to identify novel tissue-, cell-, and stage-specific gene transcripts [13,15,17-22]. Recently, several studies have investigated epididymis-specific genes using *in silico *approaches [13,15,22]; however, although these studies have provided important information about the expression profile of several epididymis-specific genes, they have been limited in the number of transcripts analyzed. By contrast, our data presented here provide systematic identification of previously uncharacterized genes with epididymis-specific or -predominant expression and further extend analysis to the cellular and biochemical level, providing insights to their potential function in sperm maturation during epididymal transit. Using information about the EST source in the database, 83 of 1505 genes were predicted to be unknown and abundantly expressed in an epididymis-specific or -predominant manner. Of these 83 possible genes, 32 were identified as authentic, epididymis-specific or -predominant genes by several expression analyses. The other 51 gene candidates were not considered further because they did not contain reliable open reading frames or coding regions, or were found to not be expressed in the epididymis or epididymis-specific by PCR analysis.

Our study provides extensive information about 32 novel genes at both the transcript and genomic levels, and 15 of these genes have also been characterized at the protein level. The Northern blot analysis, critical but usually excluded in large scale studies, revealed various characteristics of the genes at the transcript level, such as expression level, size and the presence of isoform. The genomic analysis identified an intriguing feature: the absence of orthologues in the human genome for 15 mouse genes in the human genome. Despite high synteny between the mouse and human genomes, the proportion of mouse genes with a single identifiable orthologue in the human genome is known to be about 80%. Thus, the other 20% do not have a single orthologue due to differential expansion in at least one of the two genomes. Most genes expanded in the mouse lineage have common features. These genes seem to be involved in reproduction, olfaction, and immunity, and are present as a family and found clustered in the mouse genome, suggesting that they were generated by local gene duplication. Of 25 mouse-specific gene clusters, 14 contain genes that are involved in reproduction [23]. It has been proposed that the "reproduction" genes in these clusters are related to rodent-specific aspects of reproductive physiology such as placental structures, litter sizes, estrous cycles, and gestation periods. There is a marked expansion of several families of protease inhibitors in the mouse genome compared with the human genome, similar to comparisons between the mouse and human degradomes [24,25]. Our results demonstrate that, of the 15 mouse-specific genes lacking human orthologues, three (Mm.235619, Mm.234248, and Mm.190482) are protease inhibitors. Furthermore, the recent studies on the genomic analysis of the β-defensins have reported that several β-defensins are species-specific, indicating that sequence divergence has occurred recently during evolution [14,26]. Supporting the idea that β-defensins have recently evolved by divergence and duplication, we have found no human counterpart for the four β-defensins identified in this study (Mm.99530, Mm.99387, Mm.82875, and Mm.245908), indicating either that these sequences were lost from the human genome after primate-rodent divergence, or that duplication occurred in rodents after this event.

Our study shows that most of the epididymal genes are differentially expressed in a segment-specific manner and that most genes are mainly expressed in the proximal regions of the epididymis rather than the distal regions. Furthermore, more than half of the novel genes were expressed during functional maturation of the epididymis, after the age of 16 days. Taken together, these findings suggest that many of the novel genes are expressed in epithelial secretory cells of the epididymis and have important roles in sperm maturation. Recently, the importance of proteins that are secreted in the initial segment has been confirmed by the fact that when the segment is absent, as for example in a knockout mouse for the *c-ros *tyrosine kinase receptor, the animals are sterile even though other parts of the male reproductive system are unaffected [27]. Similarly, in transgenic mice expressing the SV40 virus tumor antigen in the initial segment, the epithelium in this region is slightly hyperplastic, and its protein production is altered, resulting in infertility [28]. Thus, many novel genes that have been identified as being expressed in this region may be involved in sperm maturation or fertility, although the functional significance of these genes remains to be determined. In addition to being region-specific and developmentally regulated, epididymal gene expression is known to be affected by androgen concentrations. Consistent with this, our results have shown that many epididymal genes are regulated by androgens. Interestingly, most of the androgen-regulated genes were found to be expressed in the caput region, rather than either the corpus or cauda regions, indicating that more androgen-responsive genes are active in the caput region. Supporting this observation, several reports have shown that levels of protein synthesis are higher in the caput region than in the rest of the epididymis and the high amount of protein synthesis may be linked to androgen-activated gene expression [29].

In this study, we identified six novel epididymal genes that are predicted to encode proteins with secretory activity. UniGene information, and domain and homology searches showed that these are potential epididymal secretory protein (Mm.297297), β-defensins (Mm.99530, Mm.99387 and Mm.82875), or protease inhibitors (Mm.234248 and Mm.99782). It should be noted that, of the 32 novel genes, six were identified as β-defensins and eight contained a protease inhibitor domain (Table 2 and Figure 6). Numerous studies have shown that functionally related sets of genes often exhibit correlated patterns of gene expression and that the encoded proteins share several structural and functional characteristics [30]. Thus, it is tempting to postulate that these proteins may have similar characteristics such as secretory activity or cellular localization. Nevertheless, most of them were not expressed or, if they were expressed, were secreted. This result is consistent with previous reports suggesting that many β-defensins and protease inhibitors have cytotoxic effects as well as antimicrobial activity [16]. Recently, the rat gene Bin1b was identified and shown to be exclusively expressed in the caput region of rat epididymis. The resulting protein is responsible for sperm maturation by inducing Ca^2+ ^uptake and subsequent motility and progressive movement of immature sperm, as well as protecting sperm from infections due to antimicrobial activity [10,31]. Bin1b has structural characteristics and antimicrobial activity similar to that of β-defensins. Thus, Bin1b seems to be a natural epididymis-specific antimicrobial peptide that has roles in the reproductive tract, host defense, and male fertility. Moreover, the epididymis-specific β-defensin macaque DEFB126/ESP13.2 coats the entire ejaculated sperm and masks zona pellucida ligands on the sperm surface, but becomes dissociated when sperm are fully capacitated. This indicates that DEFB126 may be an important decapacitation factor on the sperm surface that needs to be removed before sperm-zona can interact and fertilization can occur [32,33]. It is interesting to note that six of the 32 genes in our study (Mm.190454, Mm.99530, Mm.99387, Mm.99065, Mm.82875, and Mm.245908) were identified as β-defensins [14]. Thus, it is likely that, in addition to their antimicrobial activity, each has unique functions in the epididymal tract, similar to rat Bin1b and DEFB126. However, this observation raises questions as to why these β-defensins exhibit redundancy with diverse forms and how these different proteins cooperate to protect the epididymis. Further studies are needed to fully explore the biological importance of β-defensins in the epididymis, and may lead to the development of therapeutic agents to increase immunity against sexually transmitted pathogens, and development of male infertility and contraceptive agents.

In addition to β-defensins, proteases also have important roles in several physiological processes in the epididymis. Regulation of proteases by their inhibitors is important for maintaining levels of protein degradation [34]. Previous results suggest that during maturation some of the spermatozoa modifications result from specific proteolytic processing of sperm surface proteins [35-37]. In support of the idea that proteolytic processing occurs in the epididymis, several proteases have been found in epididymal fluid [38]. Several proteases have also been found attached to the sperm surface membrane [37,39]. Hence, protease inhibitors in the epididymis might have an important role in inhibiting the activities of proteases involved in the acrosome reaction until they are needed. In addition, it has long been suggested that protease inhibitors could be involved in capacitation and fertilization, and over the past few years several protease inhibitors have been identified in epididymal secretions and characterized at the molecular level [40]. For instance, male mice lacking the protease C inhibitor Serpina 5, which is usually present at high concentrations in the male reproductive tract, are infertile, apparently owing to abnormal spermatogenesis and changes in the epididymal duct [41]. Thus, the epididymal-specific protease inhibitors identified in this study may be involved in proteolytic processing on the sperm surface in the epididymis and fertilization.

## Conclusion

Identification of genes that are expressed specifically or predominantly in the epididymis, which is indicative of their specific epididymal functions, is crucial to understanding the molecular basis of sperm maturation. The present results indicate that our genome-wide approach to gene identification may provide insights into the molecular mechanisms of sperm maturation in the epididymis. Using *in silico *and *in vitro *analyses, we have identified and characterized 32 novel genes by systematic and integrative approaches, providing insights to their region-specific and developmental expression during postnatal maturation, the hormonal regulation of their expression, and their possible secretory activity. However, further studies are needed to determine if the proteins expressed by these genes can bind to sperm and to fully understand their role in the maturation and fertilizing ability of sperm. Nevertheless, the data provided by this study provide a large resource for further investigations into molecular mechanisms of the epididymis in sperm maturation, which may help us identify new targets for the development of male contraceptive or male infertility agents.

## Methods

### RT-PCR

Total RNA was isolated from various tissues, the four regions of the epididymis, and of mice of different ages, and subsequently, cDNA was synthesized by random hexamer and oligo(dT) priming using Omniscript reverse transcriptase (Qiagen). To determine the tissue distribution of gene expression, PCR experiments were performed using cDNAs from multiple tissues (such as skeletal muscle, brain, lung, heart, liver, kidney, testis, spleen, epididymis, and vas deferens) of male mice. To investigate the region-specific expression of genes, total RNA from four different regions of epididymis (the initial segment, caput, corpus, and cauda) was used for RT-PCR analysis. To analyze the gene expression at different stages of development, RT-PCR was performed using total RNA from the epididymides of mice of different ages (7, 13, 17, 20, 30, and 60 days). Gene-specific primers are listed in Table 2. PCR was performed for 30 cycles of 94°C for 30 seconds, 55°C for 30 seconds, and 72°C for 1 minute. Primers for glyceraldehyde-3-phosphate dehydrogenase were used as a control: forward primer 5'-TGA AGG TCG GAG TCA ACG GAT TTG GT-3', and reverse primer 5'-CAT GTG GGC CAT GCG GTC CAC CAC-3'.

### Northern-blot analysis

Total RNA was isolated from each tissue using a TRI reagent (Molecular Research Center, Inc.), heated at 65°C for 5 minutes, and separated in a 1.2% agarose gel containing 1.8% formaldehyde. The gels were washed extensively in water to remove formaldehyde before transfer onto a nylon membrane (Hybond-XL; Amersham Pharmacia). Each Northern blot included 10 μg of sample RNA. The blots were prehybridized for 30 minutes at 68°C in Rapid-hyb buffer (Amersham Pharmacia), followed by hybridization for 2 hours at 68°C in the presence of a cDNA probe. Probes were derived from PCR products amplified with gene-specific primers (Table 2) and labeled with [α-^32^P]dCTP (PerkinElmer Life Sciences) using the Prime-It random priming kit (Stratagene). The blots were washed four times in 2 × SSC and 0.05% SDS at room temperature for 10 minutes and twice in 0.1 × SSC and 0.1% SDS at 68°C for 10 minutes. The blots were exposed to Hyperfilm (Amersham Pharmacia) with intensifying screens at -70°C.

### RACE

To determine the transcription initiation or termination site of novel genes, 5'- or 3'-RACE was performed using the SMART™ RACE cDNA Amplification Kit (Clontech) according to the manufacturer's instructions. Briefly, first-strand cDNA synthesis was performed using 1 μg of epididymis poly(A)^+ ^RNA, the 5'/3' cDNA synthesis primer, SMART II™ oligonucleotide, and PowerScript™ reverse transcriptase. This cDNA was then PCR-amplified using a universal primer mix (included in the RACE kit) and gene-specific primers (Table 2) by 30 cycles of 5 seconds at 94°C, 10 seconds at 68°C, and 3 minutes at 72°C. The resulting PCR products were resolved on an agarose gel, and the appropriate band was excised, purified, cloned into a pCR2.1 vector (Invitrogen) and sequenced.

### Castration

Mice were separated into three treatment groups: wild type (sham operated), castrated + sesame oil, and castrated + dihydrotestosterone (DHT; Fluka). Bilateral castrations or efferent ligation were done through the abdominal route. Anesthesia was performed by an intraperitoneal injection of ketamine (100 mg/kg) and xylazine hydrochloride (30 mg/kg). After a recovery period of 7 days, all castrated mice were divided into two groups. A control group received a 100 μl injection of 90% sesame oil and 10% ethanol (v/v), whereas the second group was injected with 5 mg of DHT dissolved in 90% sesame oil and 10% ethanol (v/v) at study start and after 24 hours. All the mice were sacrificed 1 day after the last injection, and the epididymides were removed, immediately frozen in liquid nitrogen, and stored at -80°C for RNA isolation.

### Cell culture and transfection

COS-7 cells (American Type Culture Collection) were grown in Dulbecco's minimal essential medium (DMEM; Gibco) supplemented with 10% fetal bovine serum (FBS; HyClone), 100 units/ml penicillin, and 100 μg/ml streptomycin at 5% CO_2_/95% air in a humidified incubator at 37°C. Plasmid DNA transfection of COS-7 cells was performed with Lipofectamine 2000 (Invitrogen) according to the manufacturer's instructions. Assays were performed 48 hours after transfection.

### Detection of secreted proteins

A plasmid for expression of the novel genes was constructed using the pcDNA3.1-myc/His-B vector (Invitrogen). A DNA fragment encoding the complete coding sequence was prepared by PCR amplification using the specific primers (Additional data file 3). The PCR products with *BamHI*/*XhoI, HindIII/XhoI*, or *EcoRI/XhoI *cloning sites were inserted at the *BamHI/XhoI, HindIII/XhoI*, or *EcoRI/XhoI *sites of pcDNA3.1-myc/His-B, respectively. COS-7 cells were transfected with the pcDNA3.1-myc/His-B plasmid expressing putative UniGene proteins with a myc/His epitope tag at the carboxy terminus using Lipofectamine 2000 following the manufacturer's protocol. Culture media and cells were collected by aspiration and trypsinization, respectively. UniGene proteins with a myc/His tag were immunoprecipitated with an anti-myc monoclonal antibody (9B11; Cell Signaling Technology). Immunoprecipitated proteins were separated by 15–20% SDS-PAGE and transferred to a polyvinylidene fluoride (PVDF) membrane (Pall). Membranes were immunoblotted with 9B11 followed by alkaline-phosphatase-conjugated secondary antibodies (Jackson Immunoresearch). Alkaline-phosphatase activity was detected by the NBT/BCIP reaction (Promega Biotech). Cysteine-rich secretory protein 1 (CRISP1) was used as a marker for an epididymal secretory protein and primer pairs were as follows: forward primer, 5'-ATC GGA TCC GCC ACC ATG GCA TTA ATG-3'; and reverse primer, 5'-CCG CTC GAG CGG TGA ATT TTG CC-3'

### *In silico *analysis

To investigate exon-intron structures, chromosomal location, and human synteny, the cDNA sequences of the novel genes were subjected to BLAST analysis using the NCBI Mouse Genome Resource [42] and the Wellcome Trust Sanger Institute Mouse Genome Server [43] and to BLAT analysis using the UCSC Genome Informatics resource [44]. Amino acid sequences deduced from the cDNA sequences of the novel genes were analyzed using several computational bioinformatics tools. PROSITE [45], PFAM [46], and SMART [47] were used to predict the presence of various protein patterns and profiles. SignalP [48] was used to analyze and predict the presence of putative signal peptides and their cleavage sites. PSORT II [49] was used to predict protein sorting signals and intracellular or extracellular localizations.

## Authors' contributions

JO, JL, JMW and EC performed classification and selection of genes in the epididymis library. JO, EC, IP, CH, NB and HL carried out the Northern blot and RT-PCR analysis. JO performed the protein analysis. DHK and CC conceived and directed the project. JO and CC designed the study and drafted the manuscript. All authors read and approved the final manuscript.

## Supplementary Material

Additional data file 1 List of known genes in the epididymis library Click here for file

Additional data file 2 List of unknown genes in the epididymis library Click here for file

Additional data file 3 List of primers for cloning of pcDNA3.1-UniGene-myc/His Click here for file

## Figures and Tables

**Table 1:** Classification of genes in the epididymis library

Genes	Number
Total entries	1505
Known (named or assigned)	205
Specific	10
Predominant	12
Widely expressed	183
Unknown	204
Specific	58
Predominant	25
Widely expressed	121
Single EST	1096

Gene entries from the epididymis UniGene library (as of September, 2004) were classified into known and unknown genes. Each category was further classified into epididymis-specific, epididymis-predominant, and widely-expressed genes. Single ESTs were counted and excluded in the present study. All of the known and unknown genes are listed in Additional data files 1 and 2, respectively.

**Table 2:** List of genes and gene-specific primers for RT-PCR

UniGene ID (GenBank ID)	Gene description^a^	PCR primers	
		
		Forward	Reverse
Mm.99495 (AK033813)	9230112N15Rik, hypothetical protein	GTCCGGTGCTAATAGAGCCGGCTAG	GGCTGATGAGGTCAACTGGAACTAG
Mm.190454 (AK079000)	9230107O10Rik, hypothetical protein (Defb20)	GGTTATGGGCAGTGAGTGGCACAC	GGACAACGGCCTTCGTGAACAAG
Mm.99123 (AK020307)	9230102M18Rik, hypothetical lipocalin protein (Lcn12)	CCACCACCAGCCATGCAGTTTCAG	GAGGCTCTACTGGCAGGAACCTGTTC
Mm.297297 (NM_203508)	LOC219026, hypothetical protein (Gene model 75)	AGCGACGGGTGCACTGATTAGATG	CAGAACCATCCAGAGGTGATGAGAC
Mm.99530 (AK020304)	9230102D03Rik, hypothetical protein (Defb41)	TCTTGTCCAAGAAACTGTACCATGAAG	CAGTAAGTAGTACTTCTGTGTGGCAG
Mm.99350 (AK033758)	9230104O11Rik, hypothetical protein	CATGAAAGGCTTCAGAAGAGAG	CAGAATAATAGGAATTAACACACC
Mm.235619 (AK020345)	9230113P08Rik, unknown EST	GCCGCAATGGCATGAAATCATGCTG	ACATAGAGAGGAGTATGGGGCCTG
Mm.99733 (AK020348)	9230116B18Rik, similar to ACBP	GAGTCACAGCATTCCGTGTCTCATC	GGAGATCTGATTTCTCCGTCACC
Mm.297745 (NM_001033421)	LOC330921, hypothetical protein (Gene model 846)	GGTTGCAGGATGTTTGTGCTGGTG	CAGAACTAGAGTCCCATTGGGAGG
Mm.335028 (XM_356144)	LOC382065, hypothetical protein (Gene model 1110)	GGCCGGTGGCTTCAATACTCTTACCACG	AACTTGCACGGCAATGATGGGGCCGC
Mm.229362 (AK033834)	9230117D22Rik, hypothetical protein	CACTGATCTCCAAGGCCGTGA	AGAGCTCTGCCGTCAACACCAGC
Mm.159846 (AK020329)	9230110F15Rik, hypothetical protein	ATCCCAGACTGAGATGGGCAAGC	ACTGGGACACAGTGCCATTGCTG
Mm.99387 (BY721060)	LOC432867, hypothetical protein (Defb42)	CCTTCCACCATGAGACTGTATCTGC	CCATTGCTTTAGCCGGCCGTGTGAG
Mm.99065 (AK078980)	9230002F21Rik, similar to 2D6 Gylcoprotein (Defb22)	GTTCCTGGCCCATTTGGTCACAG	GCACCAACCATTGCAGCAGTGCTGGC
Mm.82875 (AK078987)	2410125J01Rik, unknown EST (Defb30)	GTCTTGCTCTCCTATGTTCCAC	GTAGAGAACACTAGCCGGGATC
Mm.319913 (BY721150)	9230112K08Rik, unknown EST (CRISP4)	GAGTTGGAGTTCAGCTGCTGCAGAG	CACAGCCAACGAGGTAGGTAGAGGC
Mm.332572 (NM_001033459)	LOC381667, hypothetical protein (Gene model 1679)	GTCTGCTGCTGCCTTAAAGCAG	GTCCTGATGCAACAGTTGCTGTGGC
Mm.291102 (NM_001033418)	LOC330470, hypothetical protein (Gene model 767)	CAACCTGACAGCAGGAACATGGC	GGGAAAGGCACATGGAGCATAGTCTG
Mm.261496 (XM_001002680)	9230106F14Rik, hypothetical protein	CACCCGCATGACTGGTGACATCAAC	CACAGCCCCAGCTTTGGAATAGG
Mm.252404 (NM_177813)	C630025C03, hypothetical protein	CAGATGTCTCATCTTTCCCTTC	ATCAGATAATGAAAGTCCAGGGC
Mm.234248 (BB625488)	LOC629747, similar to Eppin	ACCATGCAGCTCCAGGCCTACTTC	GGCTTGACAAGTCAGGTGTTGGTG
Mm.159975 (AK020314)	9230104L09Rik, hypothetical protein (Cystatin E2)	ATGTCCAGGGAGCTCAGGCATGG	GAGCCTGGGCTGCTGATGCTG
Mm.245908^b ^(AK079042)	9230118I06Rik, hypothetical protein (Defb44)	GACCCTCCACAGCTATGAACC	CTGGAGCTGTGAGGCTAGGTC
Mm.117440 (XM_484760)	LOC433181, hypothetical protein	GTTGCAGAGTCTGCTGTTGC	GCCACCAGTTGAGAACATTCC
Mm.99782 (AK020352)	9230117E20Rik, hypothetical serine protease inhibitor	GCATGTTCAACGCCCCTAAC	GTTTGGCAGAATGCACAGCGG
Mm.190482 (AK020324)	9230107M04Rik, unclassifiable	CATCCTCCAGAACAAGTTG	GTTAGGAGAACATTGCTTCC
Mm.99576 (AK033776)	9230106D23Rik, hypothetical protease protein (Ovch2)	GTTTGTGAGGCCTGTGTGTC	CACTCCAGCCAGAGTCCAGG
Mm.99499 (AK033743)	9230101D24Rik, hypothetical lipocalin protein (Lcn6)	ATCTTTCCTAGGCCAGGCGG	TTCTGCAGCTGAGCCTGCTG
Mm.293365 (NM_001033240)	LOC209351, similar to WAP four-disulfide core 6-like 1	GAGCATCCAGGAACCTGAGC	CAGACAACGGTGCAGATACC
Mm.190489 (BY720992)	9230010P13Rik, unknown EST	AGACTACAAACCACAGCAGC	CAGTAAGTCCAGCAGCACG
Mm.99690^b ^(AK079019)	9230111O07Rik, unclassifiable	TCATGAAGCCCTCGTGGTTC	CCAGCGGGAAGTCAGGGTC
Mm.99400 (NM_001034871)	LOC328788, hypothetical protein (Gene model 749)	CACAGCGACAGGGACTTATC	GCCAGATCGACAGGGACAC

^a ^Genes annotated or named during the updates of the library are shown in parentheses.

^b ^UniGene ID numbers of Mm.245908 and Mm.99690 have been changed into Mm.387101 and Mm.389336, respectively, during the update of the UniGene database.
