# Supplementary material for: Systematic identification and integrative analysis of novel genes expressed specifically or predominantly in mouse epididymis
Source: BMC Genomics. 2006 Dec 13;7:314. doi: 10.1186/1471-2164-7-314 (PMC1764739; doi:10.1186/1471-2164-7-314)
Supplement: Additional data file 1 — List of known genes in the epididymis library [file 1471-2164-7-314-S1.pdf]

Additional data file 1
List of known genes

UniGene ID	Gene description
**Epididymis-specific genes**	
Mm.281961	defensin beta 12
Mm.99528	Rnase 9
Mm.99441	epididymal protein (Av381130)
Mm.160060	Spint4
Mm.1286	seminal vesicle protein 2
Mm.229357	defensin beta 6
Mm.99395	seminal vesicle protein, secretion 2
Mm.3787	seminal vesicle secretion 6
Mm.260341	seminal vesicle secretion 3
Mm.117462	G protein-coupled receptor 82
**Epididymis-predominant genes**	
Mm.16781	cysteine-rich secretory protein 1
Mm.1332	glutathione peroxidase 5
Mm.99511	epididymal protein Av381126
Mm.318775	Rnase 10
Mm.12867	Lipocalin 5
Mm.99424	cystatin 11
Mm.42209	tectorin alpha
Mm.117450	a disintegrin and metalloprotease domain 28
Mm.261495	a disintegrin and metalloprotease domain 7
Mm.183909	a disintegrin and metalloprotease domain 28
Mm.99556	cyclic nucleotide gated channel alpha 4
Mm.57030	phenylethanolamine-N-methyltransferase
**Ubiquitous genes**	
Mm.306720	complement component 4 binding protein
Mm.4266	integral membrane protein 2B
Mm.279485	ribonuclease/angiogenin inhibitor 1
Mm.30300	CEA-related cell adhesion molecule 10
Mm.302013	ras homolog gene family, member G
Mm.27915	nucleoredoxin
Mm.271891	sorting nexin 1
Mm.221403	platelet derived growth factor receptor, alpha polypeptide
Mm.21936	RAB21, member RAS oncogene family
Mm.14460	aldo-keto reductase family 1, member B7
Mm.9086	solute carrier family 16 (monocarboxylic acid transporters), member 1
Mm.9052	progesterone receptor membrane component 1
Mm.87639	high mobility group box transcription factor 1
Mm.46705	serologically defined colon cancer antigen 1
Mm.4172	stem-loop binding protein
Mm.38155	actin related protein 2/3 complex, subunit 5-like
Mm.34171	prominin 2
Mm.300628	synaptogyrin 2
Mm.295898	Aldehyde dehydrogenase family 6, subfamily A1
Mm.29346	guanosine monophosphate reductase 2
Mm.292547	prostaglandin-endoperoxide synthase 2
Mm.291624	anaphase promoting complex subunit 2
Mm.282053	ribosomal protein L7
Mm.280068	chromosome condensation 1-like
Mm.274926	embigin
Mm.25181	zinc finger, CCHC domain containing 11
Mm.248827	calnexin
Mm.248237	COX15 homolog, cytochrome c oxidase assembly protein (yeast)
Mm.24130	CD52 antigen
Mm.215034	matrin 3
Mm.210352	splicing factor, arginine/serine-rich 10 (transformer 2 homolog, Drosophila)
Mm.200608	Clusterin
Mm.182377	UDP-Gal:betaGlcNAc beta 1,4-galactosyltransferase, polypeptide 4
Mm.158251	plasma glutamate carboxypeptidase
Mm.143689	acyl-CoA synthetase long-chain family member 4
Mm.127681	ataxia, cerebellar, Cayman type homolog (human)
Mm.99593	transcription termination factor, mitochondrial-like

UniGene ID	Gene description
**Ubiquitous genes (continued)**	
Mm.877	chemokine (C-X-C motif) ligand 10
Mm.86705	eukaryotic translation initiation factor 2C, 4
Mm.86343	TAF3 RNA polymerase II, TATA box binding protein (TBP)-associated factor
Mm.850	signal recognition particle 14
Mm.84073	Bcl2-associated athanogene 3
Mm.826	ras homolog gene family, member Q
Mm.74711	nuclear receptor interacting protein 1
Mm.7445	MAP/microtubule affinity-regulating kinase 1
Mm.6967	Bcl2-like 2
Mm.65306	ADAMTS-like 3
Mm.641	activating transcription factor 4
Mm.6118	sorting nexin 17
Mm.582	fatty acid binding protein 4, adipocyte
Mm.5731	glutathione S-transferase, theta 3
Mm.56935	potassium voltage-gated channel, Shal-related family, member 1
Mm.549	interferon gamma receptor
Mm.5246	peptidylprolyl isomerase A
Mm.5222	serine/arginine repetitive matrix 2
Mm.47384	C-type (calcium dependent, carbohydrate recognition domain) lectin, superfamily member 6
Mm.46754	solute carrier family 38, member 2
Mm.4504	Gap junction membrane channel protein alpha 1 (Gja1)
Mm.44876	tripartite motif protein 2
Mm.444	inhibitor of DNA binding 1
Mm.42202	thrombospondin type 1 domain containing gene
Mm.41757	gamma-glutamyltransferase-like 3
Mm.40036	proline-rich polypeptide 3
Mm.3990	suppressor of K+ transport defect 3
Mm.39371	potassium voltage-gated channel, subfamily Q, member 5
Mm.392	indoleamine-pyrrole 2,3 dioxygenase
Mm.3879	hypoxia inducible factor 1, alpha subunit
Mm.38674	synaptotagmin-like 4
Mm.38241	interleukin-1 receptor-associated kinase 1
Mm.37213	protein tyrosine phosphatase, receptor type, B
Mm.3705	procollagen-proline, 2-oxoglutarate 4-dioxygenase (proline 4-hydroxylase), alpha II polypeptide
Mm.355714	melanoma antigen, family D, 1
Mm.34871	inhibitor of DNA binding 2
Mm.347639	F-box and leucine-rich repeat protein 10
Mm.347564	zinc finger, DHHC domain containing 14
Mm.347308	Metal response element binding transcription factor 2
Mm.332474	G-rich RNA sequence binding factor 1
Mm.331392	mitogen-activated protein kinase kinase 1 interacting protein 1
Mm.331051	guanine monphosphate synthetase
Mm.330501	mitogen activated protein kinase binding protein 1
Mm.328945	A kinase (PRKA) anchor protein 8
Mm.32842	natural killer tumor recognition sequence
Mm.32835	solute carrier family 25 (mitochondrial oxodicarboxylate carrier), member 21
Mm.32801	Stam binding protein
Mm.322018	activating transcription factor 7 interacting protein
Mm.3118	ARP1 actin-related protein 1 homolog A (yeast)
Mm.305047	dipeptidylpeptidase 6
Mm.30217	calcium and integrin binding 1 (calmyrin)
Mm.30199	casein kinase 1, epsilon
Mm.300594	surfeit gene 4
Mm.30010	actin related protein 2/3 complex, subunit 1B
Mm.29966	polypyrimidine tract binding protein 2
Mm.29924	ADP-ribosylation factor-like 6 interacting protein 1
Mm.297883	chloride channel 4-2
Mm.297192	ADP-ribosylation factor guanine nucleotide-exchange factor 2 (brefeldin A-inhibited)
Mm.296985	chaperonin subunit 4 (delta)
Mm.296457	pellino 2

Additional data file 1 *(continued)*

UniGene ID	Gene description
Ubiquitous genes (continued)	
Mm.296409	phosphatidylinositol-4-phosphate 5-kinase, type 1 beta
Mm.296022	membrane metallo endopeptidase
Mm.29495	CUG triplet repeat, RNA binding protein 1
Mm.294753	LPS-induced TN factor
Mm.29389	keratin complex 2, basic, gene 8
Mm.290421	early growth response 2
Mm.290126	fucosidase, alpha-L- 1, tissue
Mm.289936	phosphoserine aminotransferase 1
Mm.289739	slit homolog 2 (Drosophila)
Mm.247956	sushi domain containing 2
Mm.289082	WD repeat domain 26
Mm.28853	pituitary tumor-transforming 1 interacting protein
Mm.28768	prostaglandin E synthase
Mm.2863	intergral membrane protein 1
Mm.28614	ring finger protein 149
Mm.28520	Sloan-Kettering viral oncogene homolog
Mm.282242	scavenger receptor class B, member 1
Mm.282096	elongation of very long chain fatty acids (FEN1/Elo2, SUR4/Elo3, yeast)-like 1
Mm.28162	nucleoporin 210
Mm.280199	sphingosine-1-phosphate phosphatase 1
Mm.280125	v-crk sarcoma virus CT10 oncogene homolog (avian)
Mm.277480	mortality factor 4 like 1
Mm.275728	general transcription factor II A, 1
Mm.27567	TBC1 domain family, member 17
Mm.27560	ubiquitin-like 1 (sentrin) activating enzyme E1B
Mm.274956	six transmembrane epithelial antigen of prostate 2
Mm.274346	protein tyrosine kinase 9-like (A6-related protein)
Mm.274093	arachidonate lipoxygenase, epidermal
Mm.271578	zinc and ring finger 1
Mm.270511	transcription elongation regulator 1 (CA150)
Mm.270283	ribosomal protein S27
Mm.26870	PHD finger protein 6
Mm.262859	ring finger protein 38
Mm.261333	signal-induced proliferation-associated 1 like 1
Mm.259751	chloride channel 3
Mm.258927	eukaryotic translation elongation factor 1 delta (guanine nucleotide exchange protein)
Mm.256809	zinc finger protein 148
Mm.252210	serine (or cysteine) proteinase inhibitor, clade B, member 6a
Mm.250256	ectonucleotide pyrophosphatase/phosphodiesterase 2
Mm.2475	glutaryl-Coenzyme A dehydrogenase
Mm.246803	SWI/SNF related, matrix associated, actin dependent regulator of chromatin, subfamily a, member 5
Mm.244912	3'-phosphoadenosine 5'-phosphosulfate synthase 1
Mm.24452	enoyl Coenzyme A hydratase, short chain, 1, mitochondrial
Mm.244003	sciellin
Mm.242646	ubiquitin specific protease 9, X chromosome
Mm.239498	potassium channel tetramerisation domain containing 11
Mm.235081	BH3 interacting domain death agonist
Mm.234641	RAR-related orphan receptor beta
Mm.233117	pyrroline-5-carboxylate synthetase (glutamate gamma-semialdehyde synthetase)
Mm.22708	serine (or cysteine) proteinase inhibitor, clade H, member 1
Mm.222825	thioredoxin domain containing 7
Mm.219685	phosphatidylinositol glycan, class F
Mm.21874	proteasome (prosome, macropain) subunit, beta type 3
Mm.215110	ARP8 actin-related protein 8 homolog (S. cerevisiae)
Mm.213025	actin, alpha 2, smooth muscle, aorta
Mm.213016	G protein-coupled receptor 64
Mm.21109	gelsolin
Mm.2103	cyclin G1
Mm.210018	pleckstrin homology domain containing, family C (with FERM domain) member 1

UniGene ID	Gene description
Ubiquitous genes (continued)	
Mm.209175	myeloid/lymphoid or mixed lineage-leukemia translocation to 10 homolog (Drosophila)
Mm.207619	IQ motif containing GTPase activating protein 1
Mm.206642	mannoside acetylglucosaminyltransferase 2
Mm.205266	acetyl-Coenzyme A acyltransferase 1
Mm.20216	galactokinase 2
Mm.20047	ADP-ribosylarginine hydrolase
Mm.196634	ATP-binding cassette, sub-family C (CFTR/MRP), member 1
Mm.193539	histone 1, H1c
Mm.191749	phosphodiesterase 4A, cAMP specific
Mm.18603	histidine decarboxylase
Mm.181278	vacuolar protein sorting 24 (yeast)
Mm.178524	ubiquitin specific protease 32
Mm.173286	transportin 1
Mm.160386	ABO blood group (transferase A, alpha 1-3-N-acetylgalactosaminyltransferase, transferase B, alpha 1-3-galactosyltransferase)
Mm.153684	SERTA domain containing 1
Mm.144978	non-catalytic region of tyrosine kinase adaptor protein 2
Mm.142822	Ewing sarcoma homolog
Mm.138832	H3 histone, family 3A
Mm.13787	ceruloplasmin
Mm.124728	aarF domain containing kinase 4
Mm.12459	ankyrin repeat domain 10
Mm.121265	proteasome (prosome, macropain) subunit, alpha type 1
Mm.1161	zinc finger protein 185
Mm.1114	galactosidase, alpha
Mm.10724	ets homologous factor
Mm.105309	PDZ domain containing 6
Mm.10229	FUS interacting protein (serine-arginine rich) 1
