# Supplementary material for: Systematic identification and integrative analysis of novel genes expressed specifically or predominantly in mouse epididymis
Source: BMC Genomics. 2006 Dec 13;7:314. doi: 10.1186/1471-2164-7-314 (PMC1764739; doi:10.1186/1471-2164-7-314)
Supplement: Additional data file 2 — List of unknown genes in the epididymis library [file 1471-2164-7-314-S2.pdf]

Additional data file 2
List of unknown genes

UniGene ID	Gene description
**Epididymis-specific genes**	
Mm.99495	RIKEN 9230112N15 gene
Mm.99123	RIKEN cDNA 9230102M18 gene
Mm.117440	Transcribed sequences
Mm.235619	RIKEN cDNA 9230113P08 gene
Mm.190482	RIKEN cDNA 9230107M04 gene
Mm.99740	Transcribed sequence
Mm.99576	RIKEN cDNA 9230106D23 gene
Mm.99385	Transcribed sequences
Mm.99350	clone:9230104O11 product:hypothetical
Mm.335701	proteinTranscribed sequences
Mm.335028	Gene model 1111
Mm.297745	Gene model 846
Mm.190489	Transcribed sequences
Mm.159846	RIKEN cDNA 9230110F15 gene
Mm.99796	clone:9230118C10 product:unclassifiable
Mm.99783	clone:9230117H04 product:unclassifiable
Mm.99741	clone:9230116F02 product:unclassifiable
Mm.99690	clone:9230111O07 product:unclassifiable
Mm.99687	clone:9230111N06 product:unclassifiable
Mm.99681	clone:9230111L08 product:unclassifiable
Mm.99673	clone:9230111E06 product:unclassifiable
Mm.99596	RIKEN cDNA 9230106L01 gene
Mm.99482	Transcribed sequences
Mm.99387	Transcribed sequences
Mm.99065	RIKEN cDNA 9230002F21 gene
Mm.342795	clone:9230109L20 product:unclassifiable
Mm.342752	clone:9230101M15 product:unclassifiable
Mm.339894	clone:9230116M18 product:hypothetical protein
Mm.335058	clone:9230101G17 product:unclassifiable
Mm.331852	clone:9230110N05 product:unclassifiable
Mm.325846	clone:9230117L06 product:unclassifiable
Mm.324389	clone:9230119N23 product:unclassifiable
Mm.324096	clone:9230119M08 product:hypothetical protein
Mm.322630	clone:9230113A21 product:hypothetical protein
Mm.321613	clone:9230116G16 product:unclassifiable
Mm.320112	clone:9230112B19 product:unclassifiable
Mm.319913	Transcribed sequence with weak similarity to protein sp:P54107 Cysteine-rich secretory protein-1 precursor
Mm.319090	clone:9230111H06 product:unclassifiable
Mm.317093	clone:9230107K11 product:unknown EST
Mm.316775	clone:9230101D07 product:unclassifiable
Mm.316040	clone:9230106G15 product:unclassifiable
Mm.312513	clone:9230105G02 product:unclassifiable
Mm.310386	clone:9230002O15 product:unclassifiable
Mm.291102	Gene model 767
Mm.261512	RIKEN cDNA 9230108I15 gene
Mm.252429	Transcribed sequences
Mm.246757	RIKEN cDNA 9230106D20 gene
Mm.246591	hypothetical protein 9230109N16
Mm.234248	Transcribed sequence
Mm.229362	clone:9230117D22 product:hypothetical protein
Mm.213029	clone:9230111G17 product:unclassifiable
Mm.184185	clone:9230116O15 product:unclassifiable
Mm.182366	clone:9230118O19 product:unclassifiable
Mm.117487	clone:9230112D20 product:unclassifiable
Mm.117483	RIKEN cDNA 9230112D13 gene
Mm.117476	clone:9230109C12 product:hypothetical protein
Mm.117464	RIKEN cDNA 9230105E05 gene
Mm.117455	clone:9230104K02 product:unclassifiable
**Epididymis-predominant genes**	
Mm.190454	RIKEN cDNA 9230107O10 gene
Mm.297297	LOC219026, clone:9230103K23 product:similar to Epididymal secretory protein 3 precursor [Rattus norvegicus]
Mm.245908	RIKEN cDNA 2700070H01 gene

UniGene ID	Gene description
**Epididymis-predominant genes (continued)**	
Mm.99782	RIKEN cDNA 9230117E20 gene
Mm.99530	RIKEN cDNA 9230102D03 gene
Mm.99733	RIKEN cDNA 9230116B18 gene
Mm.99517	RIKEN cDNA D930038D03 gene
Mm.76718	cDNA sequence AJ554213
Mm.293365	Gene model 122, (NCBI)
Mm.159975	RIKEN cDNA 9230104L09 gene
Mm.117490	clone:9230112O09 product:unknown ESTRIKEN cDNA
Mm.99811	9230118O15 gene
Mm.99798	clone:9230118D16 product:unknown ESTRIKEN cDNA
Mm.99499	RIKEN cDNA 9230101D24 gene
Mm.82875	RIKEN cDNA 2410125J01 gene
Mm.44986	RIKEN cDNA 9230102K24 gene
Mm.332572	Gene model 1679, (NCBI)
Mm.261496	RIKEN cDNA 9230106F14 gene
Mm.260883	RIKEN cDNA 9230105K17 gene
Mm.252404	hypothetical protein C630025C03
Mm.221512	clone:9230109H06 product:unknown EST
Mm.215346	clone:9230112I12 product:unknown EST
Mm.200622	RIKEN cDNA 1700122C07 gene
Mm.117489	clone:9230112E12 product:unknown EST
Mm.99400	Gene model 749, (NCBI)
**Ubiquitous genes**	
Mm.312675	hypothetical protein A230091H23
Mm.99613	RIKEN cDNA C630041L24 gene
Mm.330764	RIKEN cDNA 9230106L14 gene
Mm.282122	DNA segment, Chr 7, Wayne State University 128
Mm.86373	RIKEN cDNA 2010323F13 gene
Mm.6890	RIKEN cDNA 2010107K23 gene
Mm.350950	Transcribed sequences
Mm.347997	RIKEN cDNA 4833408A19 gene
Mm.330045	RIKEN cDNA 1190006A08 gene
Mm.31626	RIKEN cDNA 4432416J03 gene
Mm.29181	RIKEN cDNA 2500002K03 gene
Mm.248843	RIKEN cDNA 3110040D16 gene
Mm.213114	RIKEN cDNA 9130423L19 gene
Mm.99850	RIKEN cDNA E130014J05 gene
Mm.99790	cDNA sequence BC062109
Mm.99727	clone:9230116A06 product:unknown EST
Mm.99648	RIKEN cDNA A430065P19 gene
Mm.99632	clone:9230108H24 product:unknown EST
Mm.99036	LOC381220
Mm.9870	RIKEN cDNA 2610528M18 gene
Mm.89828	RIKEN cDNA 5830472M02 gene
Mm.87329	RIKEN cDNA 5930437A14 gene
Mm.83634	RIKEN cDNA 6820443O06 gene
Mm.70872	clone:9230108E01 product:unknown EST
Mm.67073	RIKEN cDNA A230035L05 gene
Mm.6055	expressed sequence C81234
Mm.59812	RIKEN cDNA 1200009B18 gene
Mm.46766	RIKEN cDNA 2610528E23 gene
Mm.44213	RIKEN cDNA 1810073E21 gene
Mm.38877	RIKEN cDNA 9630015D15 gene
Mm.355161	RIKEN cDNA A530054J02 gene
Mm.353627	RIKEN cDNA 3222401L13 gene
Mm.353072	RIKEN cDNA 7530420F21 gene
Mm.347773	Transcribed sequences
Mm.347586	CDNA sequence BC057079
Mm.345095	RIKEN cDNA 9230112E08 gene
Mm.34359	RIKEN cDNA 2310068J10 gene
Mm.342856	Transcribed sequence with moderate similarity to protein ref:NP_286085.1 (E. coli) beta-D-galactosidase
Mm.342375	clone:9230110P17 product:unknown EST
Mm.341955	Gene model 1878, (NCBI)
Mm.335338	RIKEN cDNA 4922501C03 gene

Additional data file 2 ***(continued)***

UniGene ID	Gene description
Ubiquitous genes (continued)	
Mm.335582	Transcribed sequence with moderate similarity to protein ref:NP_057006.1 (H.sapiens) NAD
Mm.330809	RIKEN cDNA 9230111E07 gene
Mm.327678	Transcribed sequences
Mm.32656	RIKEN cDNA 2510006C20 gene
Mm.32615	RIKEN cDNA 2010200O16 gene
Mm.32566	RIKEN cDNA D730046L02 gene
Mm.321371	RIKEN cDNA C130037N17 gene
Mm.320183	Transcribed sequence with strong similarity to protein sp:P17008 (H.sapiens) RS16_HUMAN 40S ribosomal protein S16
Mm.314835	clone:9230111O20 product:unclassifiableRIKEN cDNA
Mm.31236	2810441K11 gene
Mm.31198	Expressed sequence AI585793
Mm.311752	Transcribed sequences
Mm.311575	RIKEN cDNA 9230116L04 gene
Mm.31129	cDNA sequence BC055447
Mm.306805	RIKEN cDNA 3100004P22 gene
Mm.304207	Similar to deleted in malignant brain tumors 1 isoform c precursor
Mm.303528	hypothetical protein 9230110J10
Mm.301939	RIKEN cDNA 4732416N19 gene
Mm.301655	RIKEN cDNA 3110004O18 gene
Mm.297443	RIKEN cDNA 9230115A19 gene
Mm.294770	RIKEN cDNA 1500011L16 gene
Mm.292888	clone:9930114B14 product:unknown ESTRIKEN cDNA
Mm.291979	9530068E07 gene
Mm.28626	RIKEN cDNA 2410018G23 gene
Mm.28597	DNA segment, Chr 15, ERATO Doi 747
Mm.285452	Expressed sequence AI663975
Mm.28437	RIKEN cDNA 2700038L12 gene
Mm.283914	RIKEN cDNA 4833412N02 gene
Mm.28205	RIKEN cDNA 2310035C23 gene
Mm.281887	RIKEN cDNA 2610020H15 gene
Mm.279158	RIKEN cDNA 2610319K07 gene
Mm.275754	Transcribed sequences
Mm.274729	RIKEN cDNA 9230110G02 gene
Mm.272616	RIKEN cDNA 2010321M09 gene
Mm.271988	RIKEN cDNA 4930403J22 gene
Mm.268922	RIKEN cDNA E030041M21 gene
Mm.264680	RIKEN cDNA 1810049K24 gene
Mm.26150	RIKEN cDNA 9230115E21 gene
Mm.260557	cDNA sequence BC057552
Mm.259276	RIKEN cDNA 9230114K14 gene
Mm.259010	RIKEN cDNA 9230105E10 gene
Mm.255784	RIKEN cDNA 2810413I22 gene
Mm.255350	RIKEN cDNA 1700041C02 gene
Mm.252421	RIKEN cDNA 4921521J11 gene
Mm.250438	RIKEN cDNA 2310046G15 gene
Mm.247533	RIKEN cDNA A430105I19 gene
Mm.247113	RIKEN cDNA A230103N10 gene
Mm.24576	RIKEN cDNA B230106I24 gene
Mm.24356	RIKEN cDNA 9130427A09 gene
Mm.23671	RIKEN cDNA 1500031H04 gene
Mm.23242	RIKEN cDNA 4933428A15 gene
Mm.227929	RIKEN cDNA 9230110F11 gene
Mm.22782	clone:9230001K01 product:unknown EST
Mm.22242	Expressed sequence AW112037
Mm.219183	RIKEN cDNA C130032F08 gene
Mm.219149	RIKEN cDNA 2810013C04 gene
Mm.215745	RIKEN cDNA 9230110M18 gene
Mm.215255	RIKEN cDNA D730039F16 gene
Mm.21187	RIKEN cDNA 1110005F07 gene
Mm.211654	hypothetical protein MGC6357
Mm.200231	expressed sequence AW049829
Mm.191894	Hypothetical protein 6430570G24
Mm.188105	RIKEN cDNA 2300006M17 gene

UniGene ID	Gene description
Ubiquitous genes (continued)	
Mm.182359	RIKEN cDNA 9230117N10 gene
Mm.182293	RIKEN cDNA 4933415I03 gene
Mm.167879	RIKEN cDNA C030014K22 gene
Mm.166568	RIKEN cDNA C030018K18 gene
Mm.159903	RIKEN cDNA 9430085M18 gene
Mm.158488	RIKEN cDNA 4921522E24 gene
Mm.156724	clone:D230046H12 product:hypothetical protein
Mm.132391	hypothetical protein C230090D14
Mm.130736	clone:9230113D18 product:unknown EST
Mm.12255	RIKEN cDNA 2310042P20 gene
Mm.117475	RIKEN cDNA 9230109A22 gene
Mm.103545	cDNA sequence BC028789
Mm.103538	clone:9230114K02 product:unclassifiable
Mm.103146	clone:9230115J04 product:unknown EST
Mm.288805	expressed sequence AV216087
Mm.37332	DNA segment, Chr 16, ERATO Doi 472, expressed
Mm.289667	DNA segment, Chr 1, ERATO Doi 448, expressed
