# Supplementary material for: Systematic identification and integrative analysis of novel genes expressed specifically or predominantly in mouse epididymis
Source: BMC Genomics. 2006 Dec 13;7:314. doi: 10.1186/1471-2164-7-314 (PMC1764739; doi:10.1186/1471-2164-7-314)
Supplement: Additional data file 3 — List of primers for cloning of pcDNA3.1-UniGene-myc/His [file 1471-2164-7-314-S3.pdf]

Additional data file 3

List of primers for cloning of pcDNA3.1-UniGene-myc/His.

UniGene ID	Forward primers	Reverse primers
Mm.190454	CCCAAGCTTGCCACCATGGGCAGTGAGTGG	CCGCTCGAGCGGATACTGATGGTGTA
Mm.297297	ATCGGATCCGCCACCATGTCCTTGGTGCCT	CCGCTCGAGCGCAGGATGGGCTCTAG
Mm.99530	ATCGGATCCGCCACCATGAAGTTTCATCTA	CCGCTCGAGCGAATGCAGCACTGGTT
Mm.99733	ATCGGATCCGCCACCATGTCCTTGCAG	CCGCTCGAGCGAATTCCATATTTC
Mm.297745	ATCGGATCCGCCACCATGTTTGTGCTG	CCGCTCGAGCGGAGTCCCATTGG
Mm.99387	ATCGGATCCGCCACCATGAGACTGTATC	CCGCTCGAGCGCTTCACCTTAGAC
Mm.82875	ATCGGATCCGCCACCATGGGGAGCCTAC	CCGCTCGAGCGCTTCACGAAGAG
Mm.234248	ATCGGATCCGCCACCATGCAGCTCCAG	CCGCTCGAGCGGGTGTTGGTGCA
Mm.159975	ATCGGATCCGCCACCATGGCTCTGCCC	CCGCTCGAGCGGAGAAGCCCCTC
Mm.245908	ATCGGATCCGCCACCATGAACCTGCGTC	CCGCTCGAGCGTATACCTTTGGTTTG
Mm.99782	ATCGGATCCGCCACCATGTTCAACGCC	CCGCTCGAGCGGCATTTCCCTTC
Mm.190482	CCCAAGCTTGCCACCATGGAAGTGATG	CCGCTCGAGCGAAAATTGTTAGG
Mm.99576	CCGGAATTCGCCACCATGCCTATCAGC	CCGCTCGAGCGATGCTTTCCACG
Mm.293365	ATCGGATCCGCCACCATGAGACTCTGG	CCGCTCGAGCGTATCCAGGAAGAC
Mm.190489	CCCAAGCTTGCCACCATGAAGCCTTGG	CCGCTCGAGCGGACTTTCATCAG
